# Autophagic Degradation of GPX4 Mediates Ferroptosis During Sheep Sperm Cryopreservation

**DOI:** 10.3390/vetsci12050490

**Published:** 2025-05-19

**Authors:** Boyuan Li, Erhan Hai, Yukun Song, Jiaxin Zhang

**Affiliations:** Inner Mongolia Key Laboratory of Sheep & Goat Genetics Breeding and Reproduction, College of Animal Science, Inner Mongolia Agricultural University, Hohhot 010018, China; qq1325345055@163.com (B.L.); haierhannb@163.com (E.H.); 13507412927@163.com (Y.S.)

**Keywords:** sperm cryopreservation, ferroptosis, GPX4, autophagy

## Abstract

Ferroptosis is implicated in cryodamage to sheep sperm, potentially due to glutathione peroxidase 4 (GPX4) degradation during freezing; however, the pathway underlying GPX4 degradation remains unclear. In this study, a comparison of cryoprotective effects between the autophagy inhibitor chloroquine (CQ) and the ubiquitination inhibitor MG132 revealed that 5 μM CQ treatment significantly enhanced the motility (*p* < 0.01) and sperm plasma membrane integrity rate (*p* < 0.01) of frozen–thawed sperm; no protective effects were observed in any MG132 treatment group. Mechanistic analysis indicated that CQ treatment substantially restored GPX4 protein expression (*p* < 0.01), and concurrently reduced lipid peroxidation (*p* < 0.01) and free iron ion accumulation (*p* < 0.01), in frozen–thawed sperm. These findings suggest that GPX4 degradation during cryopreservation occurs via the autophagy pathway. This study established a ferroptosis–GPX4–autophagy axis during sheep sperm cryopreservation and identified autophagy-mediated GPX4 loss as a potential target for enhancing sperm cryoprotection.

## Simple Summary

This study explored whether glutathione peroxidase 4 (GPX4) degradation mediates ferroptosis through the autophagy pathway during sheep sperm cryopreservation. The autophagy inhibitor chloroquine (CQ) blocked autophagic degradation of GPX4, thereby significantly improving the motility and sperm plasma membrane integrity rate of frozen–thawed sperm, while reducing lipid peroxidation and iron ion levels. Our pioneering inhibitor-controlled experiments (CQ vs. MG132) demonstrated that GPX4 degradation during freezing specifically depends on autophagy, rather than ubiquitination; this process directly initiates ferroptosis responses. GPX4 degradation in cryopreserved sheep sperm is autophagy-dependent; CQ attenuates ferroptosis-induced damage by disrupting this autophagic process, providing a novel optimization target for sperm cryopreservation.

## 1. Introduction

Sperm cryopreservation is an effective method for long-term storage of sperm [1,2,3]. However, during cryopreservation, sperm are subjected to osmotic stress, ice crystal formation, and oxidative stress [4,5]; these exposures limit the use of frozen sperm in sheep breeding. Oxidative stress-induced regulated cell death (RCD) has emerged as a critical topic in cryodamage research [6]. Efforts to identify the types of RCD and elucidate their mechanisms during sperm cryopreservation are essential for advancing our understanding of cryodamage. Previous studies have indicated that ferroptosis, rather than apoptosis, is the predominant form of RCD during goat sperm cryopreservation [7]. Furthermore, in sheep, ferroptosis inhibition substantially enhances the quality of frozen–thawed sperm, possibly due to a mechanism involving glutathione peroxidase 4 (GPX4) degradation [8].

Ferroptosis, an oxidative and iron-dependent form of RCD, is characterized by iron-catalyzed lipid peroxidation that disrupts plasma membrane structures and leads to cell death [9]. GPX4, a central regulator of ferroptosis, reduces harmful lipid peroxides to non-toxic lipid alcohols via glutathione, thereby maintaining membrane integrity and protecting cells from ferroptotic injury [10,11,12]. Inhibition of GPX4 activity or reduction of its expression consistently induces ferroptosis [13,14,15,16]. GPX4 is ubiquitously expressed in sperm across species, including humans [17] and mice [18]. Its inactivation exacerbates mitochondrial oxidative stress, diminishes sperm motility, and heightens vulnerability to DNA oxidation and lipid peroxidation [18,19,20]. Although the anti-ferroptotic function of GPX4 is well established, the mechanisms underlying its degradation remain insufficiently defined [21]. For example, the ubiquitin–proteasome system mediates sperm maturation in mice [20], and the autophagic pathway is involved in metabolic regulation in tumor cells [16]. However, the specific molecular mechanisms by which GPX4 undergoes degradation during cryopreservation of sheep sperm—such as whether this process relies on autophagy or the ubiquitin–proteasome system—remain to be elucidated.

The ubiquitin–proteasome system (UPS) and autophagy represent the two primary protein degradation pathways in eukaryotic cells [22]. The UPS mediates selective degradation of short-lived and misfolded proteins through ubiquitin tagging; the proteasome inhibitor MG132 serves as a specific blocker of rapid ubiquitin-dependent proteolysis [23]. In contrast, autophagy sequesters cytoplasmic components—such as long-lived proteins, protein aggregates, and damaged organelles—into double-membrane autophagosomes, which subsequently fuse with lysosomes to enzymatically degrade their contents [24]. Chloroquine (CQ), a lysosomal alkalinizing agent, inhibits autophagosome–lysosome membrane fusion by increasing the pH of the lysosomal lumen, and thus arresting the terminal stage of autophagic flux [25,26]. Despite this observation of CQ’s effects, the role of protein degradation—particularly GPX4 degradation—in sheep sperm cryodamage remains undefined. Therefore, this study aimed to systematically explore how protein degradation pathways influence sperm cryotolerance by employing the UPS inhibitor MG132 and the autophagy inhibitor CQ as cryoprotective interventions. These investigations seek to uncover mechanistic insights in order to optimize sperm cryopreservation protocols.

## 2. Materials and Methods

### 2.1. Experimental Design

The impacts of different concentrations (0, 2, 5, 10, 20, and 40 μM) of the autophagy inhibitor chloroquine (CQ, CM02020, Proteintech, Rosemont IL, USA) and the ubiquitination inhibitor MG132 (HY-13259, MedChemExpress, Princeton, NJ, USA) on sheep sperm cryopreservation were evaluated. Sperm motility parameters, the sperm plasma membrane integrity rate, GPX4 protein expression, lipid peroxidation levels, and iron ion concentrations were analyzed.

### 2.2. Sperm Collection, Cryopreservation, and Thawing

On each collection day, we obtained sheep ejaculates using the artificial vagina method. Post collection, fresh semen quality was promptly evaluated via a computer-assisted sperm analysis system (IVOS II CASA, IMV Technologies, L’Aigle, France) for motility assessment and a sperm densitometer (IMV Technologies) for concentration determination. From the five sheep, only semen samples meeting strict criteria (total motility ≥ 75% and sperm concentration 2–3 × 10^9^ cells/mL) were used for experiments. Qualified samples (200 μL per sheep) collected on the same day were combined in a sterile container to form a single pooled sample, representing one biological replicate.

The diluent was prepared in accordance with a previously established protocol [27]; in addition, inhibitors at different concentrations were added, as shown in Table 1 and Table 2. The solution was filtered using a 0.22 μm filter, and subsequently stored at 4 °C.

The cryopreservation protocol entailed two primary stages: semen dilution and equilibration, followed by freezing and thawing. First, pooled semen was diluted to a sperm concentration of 2–3  × 10^8^ cells/mL using a preformulated semen diluent. The diluted sample was transferred to a 1000 mL glass beaker containing 30 °C water, which was then placed in a 4 °C thermostatic refrigerator. Using a prevalidated thermal profile from our prior research [27], the semen was cooled from 30 °C to 4 °C over 4 h under controlled conditions. Upon reaching 4 °C, the semen was aliquoted into 0.25 mL straws (IMV Technologies, France) with a pipette, sealed with a commercial sealing compound (018815, IMV Technologies, France), and equilibrated on a nitrogen vapor rack for 2 h.

For the freezing step, the nitrogen vapor rack was positioned 4 cm above the liquid nitrogen surface inside a prechilled polystyrene container (30 × 20 × 15 cm external dimensions, 2 mm wall thickness) charged with liquid nitrogen. The straws, loaded with equilibrated semen, underwent vapor-phase freezing at −120 °C for 7 min within this setup. Immediately post freezing, straws were transferred to a 50 L Cryosafe liquid nitrogen dewar for long-term storage at −196 °C. Thawing of cryopreserved samples, conducted 7 days post freezing, involved immersing straws in a 37 °C water bath for 30 s to achieve uniform rewarming.

### 2.3. Determination of Sperm Motility and Movement Parameters

Sperm motility characteristics, including total motility and progressive motility, were evaluated using a computer-assisted sperm analysis system. For frozen–thawed samples, at least five fields of view per sample were examined, and a minimum of 100 sperm cells were tracked to ensure statistical validity.

### 2.4. Detection of Sperm Plasma Membrane Integrity Rate

Sperm plasma membrane integrity was evaluated using propidium iodide (PI; ST1569, Beyotime, Haimen, China). In each group, 1 × 10^6^ sperm were centrifuged at 300× *g* for 5 min. After supernatant removal, the pellets were washed with phosphate-buffered saline (PBS). After washing, to achieve a final concentration of 2 × 10^6^ cells/mL prior to PI addition, the sperm pellet was resuspended in 495 μL of non-capacitating Biggers–Whitten–Whittingham medium (NC—BWW medium, Kansas city, MO, USA). Subsequently, 5 μL of propidium iodide (PI) was introduced into the sperm suspension. The mixture was incubated at 37 °C for 10 min in the dark to prevent photobleaching. After incubation, the cells were washed as previously described and resuspended in 200 μL NC-BWW medium to reduce damage during processing, given that NC-BWW medium supports optimal sperm physiology [28]. Flow cytometry (CytoFLEX; Beckman Coulter, Pleasanton, CA, USA) was performed using the phycoerythrin channel to assess the sperm plasma membrane integrity rate. In total, 10,000 cells per biological replicate were analyzed in each trial.

### 2.5. Detection of Sperm Lipid Peroxidation Level

Lipid peroxidation levels in each group were assessed using the BODIPY™ 581/591 C11 Lipid Peroxidation Detection Kit (D3861, Thermo Fisher Scientific, Walham, MA, USA). A 100 μL aliquot of thawed sperm from each group was centrifuged at 300× *g* for 5 min. After the supernatant had been discarded, the pellet was washed twice with an appropriate volume of PBS. Subsequently, 499.5 μL of NC-BWW medium and 0.5 μL of BODIPY C11 (10 mM) were added. The mixture underwent dark incubation at 37 °C for 10 min. Post incubation, cells were centrifuged at 300× *g* for 5 min, after which the supernatant was discarded. The cell pellet was washed once with PBS, and resuspended in 200 μL of NC-BWW medium for flow cytometric analysis. Lipid peroxidation was measured via the fluorescein isothiocyanate (FITC) channel, and 10,000 cells were analyzed per biological replicate in all tests.

### 2.6. Detection of Sperm Iron Ion Concentration

Iron ion concentrations in each group were determined using the Cellular Ferrous Ion Detection Kit (FerroOrange, F374, Kumamoto, Japan). A 50 μL aliquot of thawed sperm from each group was centrifuged at 300× *g* for 5 min. Post centrifugation, the supernatant was discarded, and the cell pellet was washed twice with PBS. Subsequently, 499 μL of PBS and 1 μL of FerroOrange (1 mM) were introduced to the pellet. The mixture underwent dark incubation at 37 °C for 30 min. Following incubation, samples were subjected to flow cytometric analysis: iron ion levels were detected via the phycoerythrin (PE) channel, with 10,000 cells analyzed per biological replicate across all tests.

### 2.7. Detection of Expression Levels of Sperm GPX4 Proteins

The expression levels of GPX4 proteins in sperm were assessed by flow cytometry, using the method described by Afkhami-Ardakani et al. [28]. After sperm samples had been thawed, they were washed with an appropriate volume of PBS and centrifuged at 300× *g* for 5 min. This step was repeated three times. The cells were then fixed in 300 μL of 4% paraformaldehyde (00-5523-00, Invitrogen, Carlsbad, CA, USA) for 30 min. After fixation, the cells were treated with membrane permeabilization solution (00-5523-00, Invitrogen), and subsequently blocked with bovine serum albumin (SL1336, Coolaber, Beijing, China). A rabbit polyclonal antibody against GPX4 (1:50; 30388-1-AP, Proteintech) and a rabbit polyclonal IgG isotype control (30000-0-AP, Proteintech) were added separately and incubated with the cells for 1 h. After the cells had been washed three times with membrane permeabilization solution, donkey anti-rabbit FITC-IgG fluorescent secondary antibodies (1:200; Alexa Fluor^®^ 488, Proteintech) were added and incubated with the cells at room temperature for 30 min in the dark. Unbound fluorescent antibodies were removed by washing with PBS. Flow cytometry was performed using a 488 nm excitation wavelength. A minimum of 10,000 cells per biological replicate were acquired, with protein expression quantified as the mean fluorescence intensity above the isotype control background.

### 2.8. Statistical Analysis

The experimental data were organized in Excel (Microsoft, Redmond, WA, USA). Homogeneity of variances was first verified using Levene’s test to ensure the assumptions of parametric analysis were met. One-way analysis of variance and significance testing were performed using the ANOVA function in SAS software (version 9.4). When variances were homogeneous, Tukey’s post hoc test was used for pairwise comparisons; in cases of heterogeneous variances, Welch’s correction was applied to maintain analytical validity. FlowJo (v 10.10, TreeStar, Woodburn, OR, USA) was used for data visualization, and graphs were generated with GraphPad Prism 7.04 (GraphPad, La Jolla, CA, USA). The results are presented as mean ± standard error. All experiments were repeated at least three times. A value of *p* < 0.05 was considered statistically significant.

## 3. Results

### 3.1. Effects of Inhibiting GPX4 Protein Degradation on Sperm Motility Parameters and Sperm Plasma Membrane Integrity Rate

Inhibitor treatments were conducted to evaluate sperm motility parameters (Table 3). Low concentrations of the autophagy inhibitor CQ significantly improved sperm motility parameters (*p* < 0.05); 5 μM CQ triggered the most pronounced enhancement. In contrast, supplementation with the ubiquitination inhibitor MG132 did not result in a significant improvement in sperm motility (*p* > 0.05).

**Figure 1:**
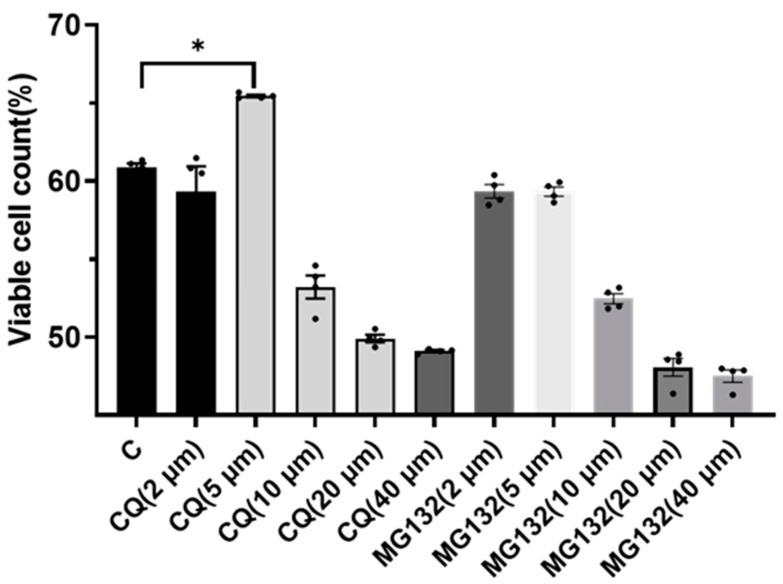
Effects of inhibitors at different concentrations on the sperm plasma membrane integrity rate of sheep sperm after freezing and thawing. Asterisks (“*”) indicate significant differences (*p* < 0.05). *n* = 4.

The sperm plasma membrane integrity rate was evaluated in parallel. As shown in Figure 1, low concentrations of CQ significantly increased membrane integrity (*p* < 0.05); 5 μM CQ demonstrated the greatest effect. MG132 supplementation did not significantly enhance the sperm plasma membrane integrity rate (*p* > 0.05).

### 3.2. Effects of Autophagy Inhibitor CQ on GPX4 Protein Expression in Frozen–Thawed Sheep Spermatozoa

**Figure 2:**
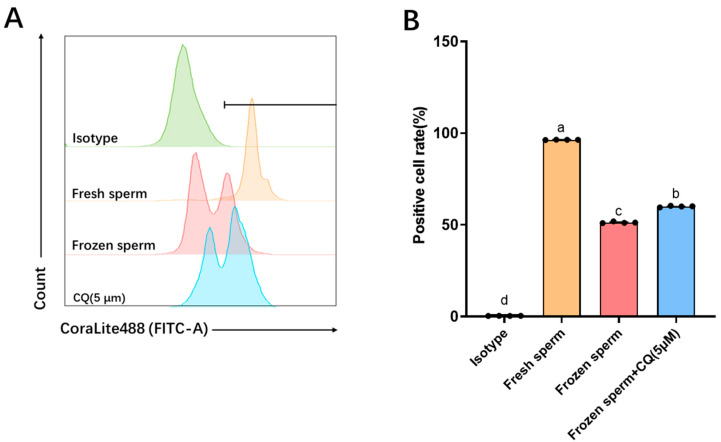
Changes in glutathione peroxidase 4 (GPX4) protein expression in sheep sperm after freezing and thawing with the autophagy inhibitor chloroquine (CQ). (**A**) Flow cytometry detection of GPX4 expression: cells gated within the “Negative” region represent positive cells. The horizontal line denotes a gate set to define the “Negative” region. (**B**) Statistical analysis of positive cell rates: bars with different lowercase letters indicate significant differences (*p* < 0.05); bars with the same lowercase letters indicate no significant differences (*p* > 0.05). *n* = 4.

As shown in Figure 2, GPX4 protein expression was evaluated in sheep sperm before and after freezing. Relative to the frozen–thawed control group, sperm treated with 5 μM CQ exhibited a significant increase in GPX4 expression (*p* < 0.05). However, GPX4 expression remained significantly lower in the CQ-treated group than in the fresh sperm group (*p* < 0.05).

### 3.3. Effects of Autophagy Inhibitor CQ on Ferroptosis Markers in Frozen–Thawed Sheep Sperm

**Figure 3:**
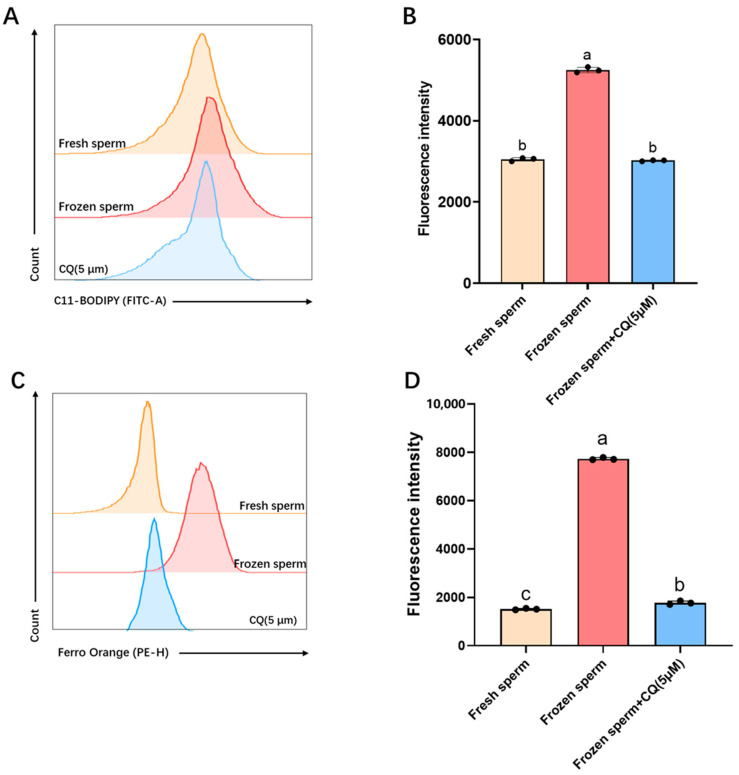
Changes in ferroptosis markers in sheep sperm after freezing and thawing with the autophagy inhibitor chloroquine (CQ). (**A**) Flow cytometry detection of lipid peroxidation levels. (**B**) Statistical analysis of the mean fluorescence intensity (MFI) of lipid peroxidation. (**C**) Flow cytometry detection of iron ion levels. (**D**) Statistical analysis of the MFI of iron ions. Bars with different lowercase letters indicate significant differences (*p* < 0.05); bars with the same lowercase letters indicate no significant differences (*p* > 0.05). *n* = 3.

The lipid peroxidation levels in sheep sperm were measured before and after freezing (Figure 3A,B). Compared with fresh sperm (3050 ± 33.95), the lipid peroxidation levels in frozen–thawed sperm were significantly elevated (5250 ± 54.79, *p* < 0.05). Treatment with 5 μM CQ significantly reduced lipid peroxidation in frozen–thawed sperm (3023 ± 11.36, *p* < 0.05).

Analysis of sperm iron ion levels (Figure 3C,D) indicated that frozen–thawed sperm exhibited significantly higher iron ion concentrations (7733.67 ± 41.59) compared with fresh sperm (2020.33 ± 48.92, *p* < 0.05). This increase was significantly attenuated by 5 μM CQ supplementation, which reduced the iron ion levels in frozen–thawed sperm to 2320.1 ± 41.59 (*p* < 0.05).

## 4. Discussion

Ferroptosis inhibition may represent a key strategy for improving the quality of frozen–thawed sperm. However, the regulatory network governing ferroptosis is highly complex; it involves redox homeostasis, as well as iron metabolism and lipid regulatory pathways. Thus, efforts to identify the central proteins that mediate ferroptosis during sheep sperm cryopreservation remain essential. As the core regulatory factor in the ferroptosis pathway, GPX4 suppresses ferroptosis by catalyzing the glutathione-mediated reduction of lipid hydroperoxides, thus efficiently neutralizing toxic lipid products [29,30]. In our previous study [8], we found that GPX4 expression was significantly lower in frozen–thawed sperm than in fresh sperm. In most cell types, the use of GPX4 inhibitors reliably induces ferroptosis [31,32]. Furthermore, in human sperm, GPX4 expression is significantly and positively associated with both motility and structural integrity; its enzymatic activity is closely associated with multiple parameters used to assess fertility [33,34]. These findings support the role of GPX4 as a key regulatory protein during sheep sperm cryopreservation. The objective of the present study was to improve the quality of frozen–thawed sheep sperm by increasing the level of the GPX4 protein. Considering that protein degradation pathways involve both autophagy and ubiquitination, various concentrations of the autophagy inhibitor CQ and the ubiquitination inhibitor MG132 were individually added to the sperm cryodiluent. Their effects on the quality of frozen–thawed sperm and the expression of the GPX4 protein were then systematically evaluated.

Autophagy, a conserved mechanism that regulates ferroptosis, is closely associated with the ferroptosis pathway [35,36]. Previous studies have demonstrated that hyperactivation of the autophagy–lysosome system can induce ferroptosis by promoting iron overload [37,38,39]. Conversely, inhibition of autophagosome formation through autophagy inhibitors or autophagy-related gene silencing can effectively suppress the production of reactive oxygen species and reduce cell death induced by oxidative stress [39,40]. In the present study, treatment with CQ significantly enhanced the motility and sperm plasma membrane integrity rate of frozen–thawed sperm, whereas MG132 treatment did not confer a protective effect. This observation aligns with findings by Mauthe et al. regarding the pharmacological actions of autophagy inhibitors [41]. A likely explanation is that the autophagy pathway remains active in human sperm and participates in the regulation of cell survival and motility [42]. A similar mechanism was identified in stallion sperm by Aparicio and colleagues, who found that CQ effectively preserved the proportion of viable sperm by inhibiting autophagy [43]. At the molecular level, CQ functions as a lysosomal lumen-alkalinizing agent [25,44], inhibiting hydrolase activity through increased lysosomal pH [26,45]. This disruption leads to lysosomal inactivation, LC3-II accumulation, and eventual blockage of autophagic flux [46,47]. Collectively, these findings suggest that abnormal activation of the autophagy pathway during sperm freezing aggravates cellular damage via the ferroptosis pathway; CQ may exert a protective effect by specifically targeting and inhibiting this process.

During sperm cryopreservation, GPX4 expression was significantly downregulated; GPX4 protein levels were positively associated with both total motility and progressive motility. These observations support our previous finding that ferroptosis inhibition reduces cryodamage in sheep sperm [8]. Importantly, recent data indicate that autophagy facilitates lipid peroxide accumulation by promoting GPX4 degradation [48], For example, chaperone-mediated autophagy of GPX4 promotes ferroptosis in renal tubules during acute kidney injury (AKI) [49], a mechanism confirmed in several ferroptosis-related pathological models [50,51]. To our knowledge, the present study is the first to show that CQ supplementation significantly increases GPX4 protein expression during sheep sperm cryopreservation. This effect likely results from suppression of the autophagy pathway, which becomes activated during freezing and accelerates GPX4 degradation, thereby promoting ferroptosis. By inhibiting autophagosome–lysosome fusion [52], CQ preserves GPX4 stability. Sustained GPX4 expression limits intracellular free iron accumulation and effectively suppresses downstream lipid peroxidation [10], resulting in targeted inhibition of sperm ferroptosis. This finding is strongly supported by the existing theoretical framework concerning autophagy-dependent regulation of ferroptosis [53,54].

Autophagy activation induced by sperm cryopreservation promotes ferroptosis by accelerating GPX4 degradation, whereas the autophagy inhibitor CQ suppresses ferroptosis by stabilizing GPX4 expression, reducing free iron accumulation, and inhibiting its catalytic role in lipid peroxidation. This study provides indirect evidence for autophagy-mediated regulation of GPX4 degradation through three key observations: cryopreserved sperm quality, GPX4 expression levels, and ferroptosis markers. However, the specific molecular pathway responsible for GPX4 autophagic degradation—including critical steps such as selective autophagy receptor recognition—was not addressed in this study. These mechanistic aspects, which are essential for understanding how autophagy selectively targets GPX4 for degradation, will be prioritized in future research.

## 5. Conclusions

This study reveals that GPX4 degradation during freezing depends on the autophagy pathway rather than the UPS, which can provide a theoretical foundation for strategies to improve sperm cryopreservation, and deliver a practical benefits to sheep production.

## Figures and Tables

**Table 1:** Formulations of CQ inhibitors at different concentrations.

Constituent	Control	CQ (2 μM)	CQ (5 μM)	CQ (10 μM)	CQ (20 μM)	CQ (40 μM)
Tris (Sigma Aldrich, St. Louis, MO, USA)	1.8 g	1.8 g	1.8 g	1.8 g	1.8 g	1.8 g
Citric acid (Sigma Aldrich, MO, USA)	1 g	1 g	1 g	1 g	1 g	1 g
Glucose (Sigma Aldrich, MO, USA)	0.5 g	0.5 g	0.5 g	0.5 g	0.5 g	0.5 g
Pen Strep (Gibco, Grand Island, NY, USA)	0.5 mL	0.5 mL	0.5 mL	0.5 mL	0.5 mL	0.5 mL
6% glycerol (Sigma Aldrich, MO, USA)	3 mL	3 mL	3 mL	3 mL	3 mL	3 mL
Egg yolk (Charoen Pokphand Group, Beijing, China)	15 mL	15 mL	15 mL	15 mL	15 mL	15 mL
CQ (Proteintech, IL, USA)	0 μM	2 μM	5 μM	10 μM	20 μM	40 μM
Total volume	50 mL	50 mL	50 mL	50 mL	50 mL	50 mL

**Table 2:** Formulations of MG132 inhibitor at different concentrations.

Constituent	Control	MG132 (2 μM)	MG132 (5 μM)	MG132 (10 μM)	MG132 (20 μM)	MG132 (40 μM)
Tris (Sigma Aldrich, MO, USA)	1.8 g	1.8 g	1.8 g	1.8 g	1.8 g	1.8 g
Citric acid (Sigma Aldrich, MO, USA)	1 g	1 g	1 g	1 g	1 g	1 g
Glucose (Sigma Aldrich, MO, USA)	0.5 g	0.5 g	0.5 g	0.5 g	0.5 g	0.5 g
Pen Strep (Gibco, NY, USA)	0.5 mL	0.5 mL	0.5 mL	0.5 mL	0.5 mL	0.5 mL
6% glycerol (Sigma Aldrich, MO, USA)	3 mL	3 mL	3 mL	3 mL	3 mL	3 mL
Egg yolk (Charoen Pokphand Group, Beijing, China)	15 mL	15 mL	15 mL	15 mL	15 mL	15 mL
MG132 (MedChemExpress, NJ, USA)	0 μM	2 μM	5 μM	10 μM	20 μM	40 μM
Total volume	50 mL	50 mL	50 mL	50 mL	50 mL	50 mL

**Table 3:** Effects of inhibitors at different concentrations on motility parameters of sheep sperm after freezing and thawing.

Group	TM(%)	PM(%)
C	59.36 ± 3.20 ^b^	27.72 ± 1.66 ^cd^
CQ (2 μm)	61.23 ± 2.86 ^b^	52.39 ± 2.84 ^a^
CQ (5 μm)	72.71 ± 4.18 ^a^	54.47 ± 4.12 ^a^
CQ (10 μm)	57.70 ± 4.56 ^bc^	41.14 ± 5.00 ^b^
CQ (20 μm)	48.64 ± 4.48 ^ef^	38.44 ± 3.83 ^b^
CQ (40 μm)	45.38 ± 3.88 ^f^	30.70 ± 3.23 ^c^
MG132 (2 μm)	53.85 ± 2.59 ^cd^	29.80 ± 4.41 ^cd^
MG132(5 μm)	58.64 ± 3.17 ^bc^	31.36 ± 4.00 ^c^
MG132 (10 μm)	51.61 ± 2.67 ^de^	26.01 ± 3.48 ^d^
MG132 (20 μm)	51.28 ± 4.38 ^de^	20.83 ± 1.66 ^e^
MG132 (40 μm)	32.29 ± 5.09 ^h^	12.89 ± 4.00 ^f^

C: control; CQ: chloroquine; TM: total motility; PM: progressive motility. Within each column, values sharing the same superscript letter are not significantly different (*p* > 0.05); values with different superscript letters are significantly different (*p* < 0.05). *n* = 5.

## Data Availability

The datasets generated and analyzed during this study are included in the article. For further inquiries, please contact the corresponding author.
